# National-Scale Conservation Gaps and Priority Areas for Invasive Plant Control in China: An Integrated MaxEnt-InVEST Framework

**DOI:** 10.3390/plants15060898

**Published:** 2026-03-13

**Authors:** Bao Liu, Mao Lin, Siyu Liu, Xingzhuang Ye, Shipin Chen

**Affiliations:** 1 College of Forestry, Fujian Agriculture and Forestry University, Fuzhou 350002, China; 13408543373@163.com (M.L.); 13881976640@163.com (S.L.); yxz@fafu.edu.cn (X.Y.); 2 Forestry and Landscape Gardening Bureau, Qingshen County, Meishan 620400, China

**Keywords:** invasive species, MaxEnt, InVEST, conservation gaps, China, climate change

## Abstract

Invasive alien plants (IAPs) pose a severe and escalating threat to biodiversity and ecosystem services in China. However, a systematic nationwide assessment that identifies invasion hotspots, quantifies their overlap with protected area networks, and pinpoints critical conservation gaps is still lacking. This hinders the development of spatially targeted management strategies. To address this, we developed an integrated analytical framework coupling the Maximum Entropy (MaxEnt) model with the InVEST habitat quality model. Using a high-resolution, county-level distribution database of 293 IAPs, we mapped potential species richness and habitat degradation across China. The geo-detector model was further employed to identify the primary environmental factors and their interactions. Spatial overlay analysis was conducted to delineate core invasion habitats (areas of high invasion suitability and high degradation) and assess their coverage within China’s national nature reserves. Nighttime light intensity (DMSP, 34.39%), annual precipitation (Bio12, 14.16%), and mean diurnal range (Bio2, 11.82%) were the factors with the highest contribution in the model, highlighting the statistical interaction between anthropogenic pressure and climatic conditions. The core invasion habitat spanned 20.10 × 10^4^ km^2^, predominantly (66.04%) concentrated in high-intensity human disturbance zones. Notably, only 11.18% of this core habitat falls within existing national nature reserves, revealing a vast conservation gap of 17.85 × 10^4^ km^2^. Our results indicate a profound spatial mismatch between invasion hotspots and the current protected area network in China. We prioritize southeastern coastal urban agglomerations-characterized by high anthropogenic pressure (DMSP), high precipitation (Bio12), and low diurnal temperature range (Bio2)-for immediate monitoring and intervention. This integrated assessment provides a national-scale, spatially explicit prediction of invasion risk for 293 plant species in China, and offers an evidence-based decision-support tool for optimizing invasive species management and biodiversity conservation.

## 1. Introduction

Biodiversity is fundamental to the Earth’s ecosystems, playing a critical role in maintaining ecosystem stability and resilience [1]. However, rapid economic development, urbanization, industrialization, and the expansion of transportation networks have accelerated biological invasions, causing severe ecosystem degradation [2]. IAPs rank among the most serious threats to global biodiversity and ecosystem integrity in the 21st century [3]. Since the mid-20th century, numerous international initiatives have been established to counter biodiversity loss [4]. Nevertheless, global assessments indicate that an additional 35.3% of terrestrial area requires protection to effectively safeguard biodiversity [5]. A survey of 69 Chinese national nature reserves documented 219 invasive alien species [6], indicating insufficient conservation capacity within existing protected areas and underscoring the need for advanced methodologies to identify and prioritize areas for biodiversity conservation [7].Predicting the distribution and impact of invasive plants is a central focus in invasion ecology. Species distribution models (SDMs) are key tools for assessing invasion risk, widely used to estimate habitat suitability by correlating species occurrence data with environmental predictors [8]. Among these, the Maximum Entropy (MaxEnt) model is prominent due to its robust performance with limited samples [9], as applied in studies of *D. ambrosioides*, *C. argentea* and *A. palmeri* genus [10], and *Phragmites australis* [11]. However, a key limitation of MaxEnt is its inadequate consideration of habitat quality and ecological resilience, which can lead to differences between predicted suitability and actual invasion success.

China is among the countries most severely impacted by biological invasions globally. According to the 2020 Bulletin on Ecological and Environmental Status in China, over 660 invasive alien species have been documented, with 277 new species recorded between 2005 and 2020 alone [12]. These invasions have been associated with substantial economic losses and persistently threaten native biodiversity and ecosystem stability. Eleven major invaders have caused cumulative economic losses estimated at USD 236.3 billion since the 1980s [12]. Despite this urgency, effective management is often hampered by a lack of precise, spatially explicit guidance. While numerous studies have predicted the potential distribution of individual or regional sets of invasive plants using SDMs like MaxEnt [13], these approaches primarily reflect environmental suitability and may overestimate risk in areas with high ecosystem resilience. Conversely, assessments of habitat quality and degradation, such as those provided by the InVEST model, identify ecologically vulnerable areas but do not directly quantify species-specific invasion pressure [14].

This disconnect highlight a critical knowledge gap: the absence of a national-scale identification of areas that are both highly suitable for invasion and under significant ecological pressure. These areas represent the “core invasion habitats” that should be the highest priority for intervention. Furthermore, the alignment between these high-priority invasion fronts and the existing network of protected areas (PAs) remains largely untested, resulting in unknown conservation gaps. A recent study on urban bird conservation has successfully integrated MaxEnt and InVEST to identify conservation priorities and gaps, illustrating the potential of this coupled approach [15]. Building on the methodological foundation, the present study applies the integrated framework at an unprecedented scale-293 invasive plant species across all of China-to examine a critical national conservation challenge while providing a methodological framework that can be applied to large-scale invasion risk assessment in other regions facing similar threats.1

**Figure 1:**
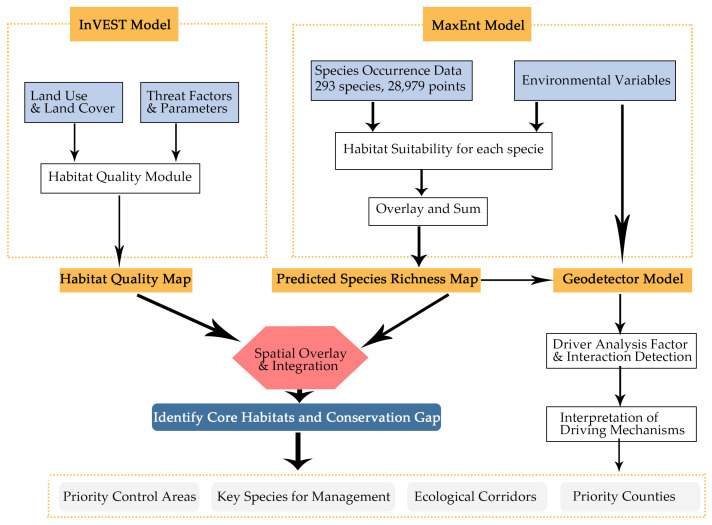
Workflow for identifying core invasion habitats by integrating the MaxEnt and InVEST models.

Therefore, this study aims to bridge this gap by conducting a comprehensive national-scale assessment of invasive plant risks and conservation needs. The specific objectives are to: (1) map the potential distribution and species richness hotspots of 293 major IAPs across China using the MaxEnt model; (2) identify the key environmental and anthropogenic drivers shaping these invasion patterns, using the geo-detector model to elucidate factor interactions; (3) evaluate the spatial pattern of habitat quality and degradation using the InVEST model; (4) integrate the results of MaxEnt and InVEST to delineate core invasion habitats; and (5) conduct a conservation gap analysis by overlapping core invasion habitats with China’s national nature reserves to identify priority areas for targeted control and management (Figure 1). The findings are expected to provide a scientifically robust, spatially explicit blueprint for optimizing national invasive species management and enhancing the efficacy of biodiversity conservation efforts.

## 2. Results

### 2.1. Key Environmental Factors Associated with Distribution

The MaxEnt model demonstrated high predictive accuracy for the 293 IAPs, with a mean area under the curve (AUC) of 0.972 (range: 0.804–1.0). These species belonged to 187 genera and 70 families. The *Asteraceae* family was the richest, containing 31 species (10.58%), followed by *Poaceae* (17 species, 5.80%). At the genus level, *Ipomoea* (10 species, 3.41%), *Amaranthus*, and *Euphorbia* (8 species each) contained the highest number of invasive species.

### 2.2. Dominant Environmental Drivers

**Figure 2:**
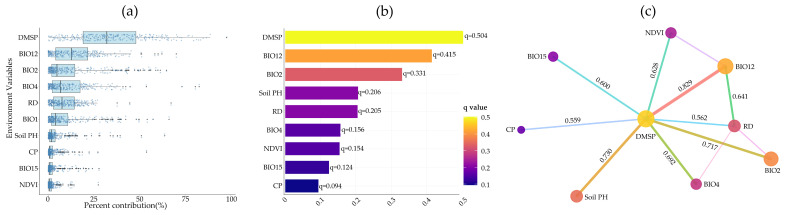
Key environmental factors of invasive plant distribution: (**a**) Relative contributions of environmental predictors to the MaxEnt model, (**b**) The q-statistic value of each factor from the Geo-detector model, quantifying its influence on the spatial heterogeneity of species richness, (**c**) Interaction effects between factor pairs.

The five environmental variables with the highest mean contribution to the MaxEnt model predictions were nighttime light intensity (DMSP, 34.39%), annual precipitation (Bio12, 14.16%), mean diurnal range (Bio2, 11.82%), temperature seasonality (Bio4, 10.91%), and road density (RD, 9.36%), collectively explaining 80.65% of the distribution patterns (Figure 2a). In contrast, precipitation seasonality (Bio15) and the normalized difference vegetation index (NDVI) showed minimal influence (<2.5% each). Factor detector analysis using the geo-detector model showed DMSP (q = 0.504) as the factor with the highest individual explanatory power in explaining the spatial heterogeneity of species richness, independently accounting for 50.4% of the variation (Figure 2b). This was followed by Bio12 (q = 0.415) and Bio2 (q = 0.331). Interaction detector analysis revealed significant nonlinear interactions between variables (Figure 2c). The most pronounced interaction was between DMSP and Soil pH (q = 0.730), where their combined explanatory power exceeded the sum of their individual effects, indicating a non-linear enhancement of spatial association in areas characterized by both high human activity and alkaline soils.

### 2.3. Spatial Patterns of Potential Species Richness

**Figure 3:**
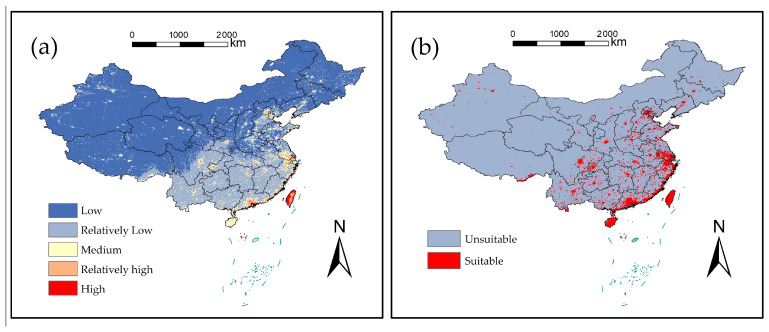
Predicted distribution and areal extent of invasive plant richness: (**a**) Spatial pattern of potential species richness of invasive plants in China predicted by the MaxEnt model, and (**b**) Proportional area of suitable and unsuitable habitats.

The predicted species richness, reclassified using the Jenks natural breaks method, showed a highly heterogeneous spatial pattern (Figure 3a). Species richness was predominantly concentrated in economically developed coastal regions, including Taiwan, Hainan, Guangdong, Shanghai, and Jiangsu. The total area of high species richness was 12.88 × 10^4^ km^2^. The total suitable habitat area for IAPs was 54.13 × 10^4^ km^2^, accounting for 5.64% of China’s land area, with the remaining 94.36% classified as unsuitable (Figure 3b). At the provincial level, suitable habitat area was most extensive in Guangdong (6.35 × 10^4^ km^2^), Zhejiang (4.19 × 10^4^ km^2^), and Jiangsu (3.49 × 10^4^ km^2^), which together comprised 66.2% of the national total. Meanwhile, eight regions-including Hong Kong (0.06 × 10^4^ km^2^) and Qinghai (0.07 × 10^4^ km^2^)-each contained less than 0.5 × 10^4^ km^2^ of suitable habitat. Notable county-level hot-spots were identified in southeastern Tibet (e.g., Cona County) and the mountainous eastern coast of Taiwan (e.g., Hualien County).

### 2.4. Habitat Quality and Degradation Patterns

**Figure 4:**
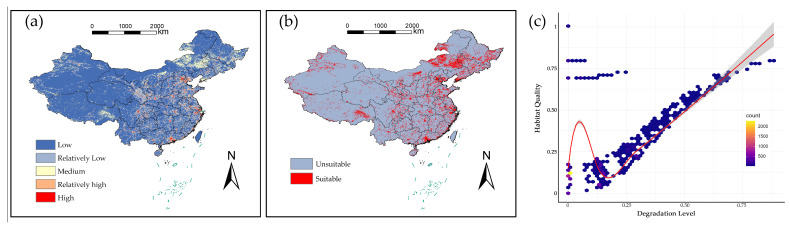
Results of the integrated modeling approach: (**a**) Predicted geographic distribution of invasive plant species richness in China, (**b**) Statistical summary of habitat suitability area, and (**c**) The significant negative correlation between habitat quality and habitat degradation pressure.

These patterns were combined into a comprehensive habitat degradation index (Figure 4a). The index, ranging from 0 to 0.93 across China, was classified into five tiers: high (0.55–0.93), relatively high (0.42–0.55), medium (0.31–0.42), relatively low (0.17–0.31), and low (0–0.17) degradation. For the subsequent integrated analysis, areas with an index value of medium or higher (≥0.31) were defined as high-vulnerability habitats (Figure 4b).

Assessment using the InVEST model revealed that habitat degradation pressure (the inverted quality index) varied widely across China, ranging from 0 to 0.93. A significant negative correlation was observed between habitat quality and this degradation pressure (Figure 4c). Spatially, wetland areas accounted for the largest proportion of the landscape (29.69%), yet exhibited the lowest degradation level (0.034), which may indicate a degree of ecological resilience. In contrast, high-intensity disturbance zones, which covered 26.52% of the area, showed the highest degradation index (0.159), marking them as the most vulnerable ecosystem type.

Among the specific threat factors analyzed, urbanization showed the highest correlation coefficient with habitat degradation (r = 0.793, *p* < 0.001). Agricultural activities (r = 0.572) and highway density (r = 0.550) were also significant contributors. Railway networks, rivers, and ports had moderate influence, while tourism activities showed limited impact.

### 2.5. Identification of Core Invasion Habitats

**Figure 5:**
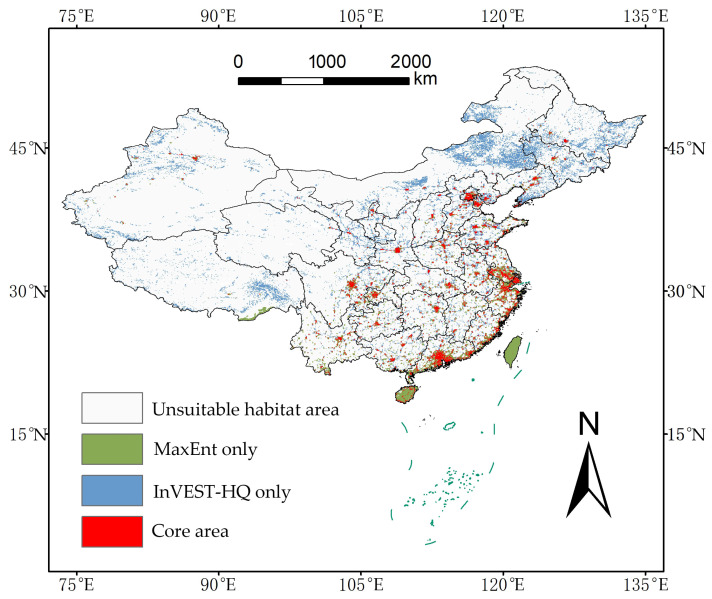
Core habitats for invasive plants identified by model integration.

The core invasion habitats were defined as the spatial intersection of areas with high suitability for IAPs (from MaxEnt) and high habitat vulnerability (from InVEST), covering 20.10 × 10^4^ km^2^ (2.51% of China’s land area). Land-cover analysis showed these core habitats were predominantly concentrated in human-modified landscapes, with 66.04% located within high-intensity disturbance zones. For context, the suitable area identified only by MaxEnt was 32.93 × 10^4^ km^2^, while the highly degraded area identified only by InVEST was 87.27 ×10^4^ km^2^. The spatial distributions of these single-model outputs are shown for comparison (Figure 5). The concentration of the integrated core area within anthropogenic environments highlights the association between human pressure and invasion risk.

### 2.6. Conservation Gaps and Priority Control Areas

**Figure 6:**
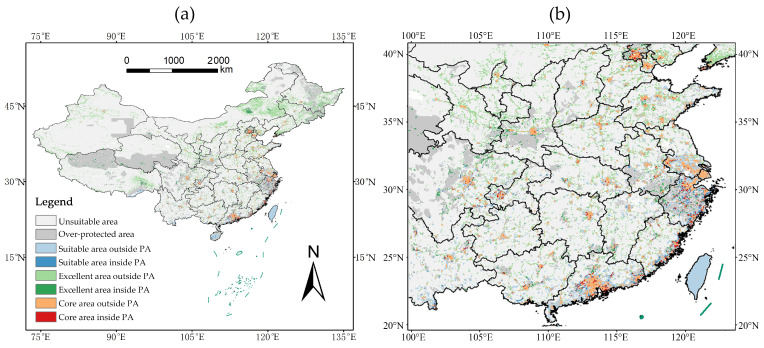
Conservation gaps and priority areas for invasive species control. (**a**) National-scale distribution of core invasion habitats and protected areas; (**b**) Enlarged view of the Pearl River Delta and Yangtze River Delta showing the spatial mismatch between invasion hotspots and protected area coverage.

A critical spatial mismatch was revealed between the identified core invasion habitats and China’s protected area (PA) network. Only 11.18% (2.25 × 10^4^ km^2^) of the total core habitat area is currently within existing PAs (Figure 6). Consequently, a substantial conservation gap of 17.85 × 10^4^ km^2^-representing 88.82% of the core habitats-lies outside the current conservation network. These gap areas show high levels of anthropogenic pressure and are geographically concentrated in southeastern coastal provinces and major urban agglomerations, such as the Pearl River Delta and the Yangtze River Delta. Based on their high invasion suitability and elevated ecosystem vulnerability, these regions constitute the highest priority for targeted invasive species control and proactive management interventions.

## 3. Discussion

### 3.1. Environmental Variables Associated with Distribution Patterns

Our integrated analysis indicates that the spatial distribution of invasive alien plants (IAPs) in China is associated with not a single factor but with interactions between anthropogenic pressure and climatic gradients. The dominance of nighttime light intensity (DMSP, 34.39% contribution in MaxEnt; q = 0.504 in geo-detector) as the foremost predictor highlights a strong statistical association between human activity and plant invasion patterns, consistent with the propagule pressure hypothesis. In some other ecosystems, the legacy effects of historical disturbances may have created conditions conducive to invasion [16]. High population density is also associated with increased soil pollution [17]. This pattern is consistent with the “propagule pressure” hypothesis, wherein transportation hubs, urban centers, and intensive agricultural zones act as primary introduction points and dispersal corridors. Our finding that 66.04% of core invasion habitats are concentrated in high-intensity disturbance zones strongly supports this hypothesis by revealing a clear spatial association between human activity and predicted invasion fronts.

More critically, the geo-detector analysis revealed nonlinear interactions between environmental factors. The strongest interaction term was between DMSP and Soil ph (q = 0.730), suggesting a particularly strong combined association in areas with both high human disturbance and alkaline soil conditions. This statistical interaction raises the hypothesis that areas experiencing both high human disturbance and alkaline soil conditions may be particularly vulnerable to invasion—potentially due to altered disturbance regimes or soil properties that favor certain functional groups. A second key interaction between annual precipitation (Bio12) and road density (RD, q = 0.641) suggests that infrastructure networks show associations with invasions in climatically favorable regions, where they act as “highways” for dispersal [18].

**Figure 7:**
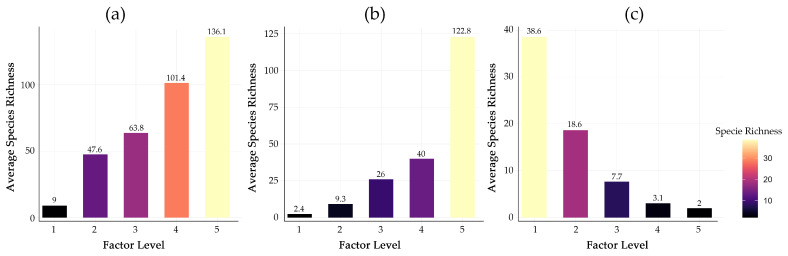
Risk distribution of the dominant environmental factors. Response of invasive plant species richness to the three most influential environmental factors: (**a**) Nighttime light intensity (DMSP), (**b**) Annual precipitation (Bio12), and (**c**) Mean diurnal temperature range (Bio2).

The response curves further illustrate the form of these statistical relationships (Figure 7). Habitat quality is closely linked to invasion success [19], reflecting resource availability within ecosystems [20], while urban disturbance promotes the growth of invasive plants [21].The monotonically increasing relationship between DMSP and species richness is consistent with the hypothesis that propagule pressure associated with human activity is a primary factor shaping invasion patterns. Species richness exhibits a nonlinear, threshold-dependent increase with Bio12, suggesting that above a certain precipitation level (2000 mm), the predicted habitat suitability for a broad spectrum of IAPs is substantially higher. Conversely, the negative correlation with Bio2 implies that thermally stable environments lower physiological barriers for nonnative species. The response curves quantify statistical relationships between environmental gradients and predicted species richness, identifying threshold-like associations that can inform hypothesis generation about underlying ecological processes.

**Figure 8:**
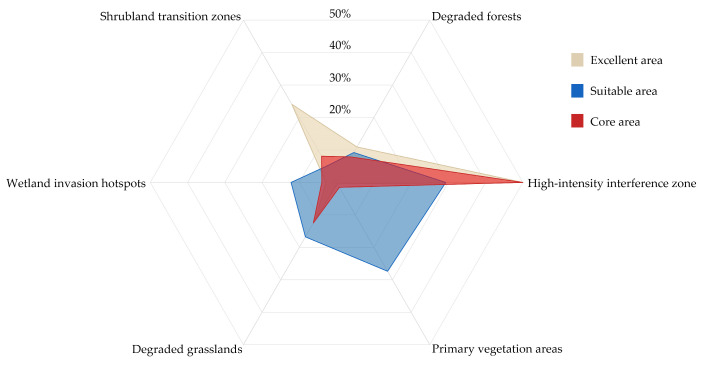
Land covers composition of priority areas. Proportional areal coverage of different land-use/land-cover (LULC) types within the core area, suitable habitat area, and high-quality habitat area.

Among the threat factors, urbanization exhibited the strongest positive correlation with habitat degradation (r = 0.793, *p* < 0.001), indicating a strong association between urban expansion and modeled habitat degradation. Agricultural activities (r = 0.572) and highways (r = 0.550) ranked second and third, respectively, highlighting the substantial associations with agricultural expansion and transportation infrastructure development (Figure 8).

### 3.2. A Profound Spatial Mismatch: Invasion Hotspots Versus Protected Areas

A key finding of this study is the severe spatial mismatch between identified invasion fronts and China’s existing conservation infrastructure. Our integration of MaxEnt (suitability) and InVEST (degradation) models identified a core invasion area of 20.10 × 10^4^ km^2^, representing landscapes that are both highly suitable for IAP establishment and ecologically degraded, hence possessing low resilience.

The gap analysis reveals that only 11.18% (2.25 × 10^4^ km^2^) of this core area falls within national nature reserves, leaving a conservation gap of 17.85 × 10^4^ km^2^ (88.82%). This gap shows a non-random spatial pattern, with higher concentrations in China’s most dynamic and economically vital regions: the southeastern coastal urban agglomerations, the Pearl River Delta, and the Yangtze River Delta. These areas show a “two-high-one-low” pattern: high anthropogenic pressure (DMSP), high precipitation (Bio12), and low diurnal temperature variation (Bio2).

This mismatch highlights a potential limitation in China’s conservation strategy. Protected areas (PAs) have traditionally been located in remote, bio-diverse regions with low human footprint. However, our results indicate that the highest predicted invasion risks are outside this network, in human-dominated landscapes where ecosystems are already stressed. The low protection rate (<12%) for core invasion habitats suggests that the current PA system may have limited coverage for areas with high predicted invasion risk. This suggests the need for a broader approach in conservation planning, extending protection and management interventions beyond traditional PA boundaries into working landscapes and urban-peri-urban interfaces.

### 3.3. Towards a Prioritized and Tiered Management Framework

Our spatially explicit maps translate ecological insights into a decision-support tool for implementing China’s “Biodiversity Conservation Strategy and Action Plan (2023–2030)” and the “Management Measures for Alien Invasive Species”. We propose a tiered and adaptive management framework that prioritizes actions based on risk level and landscape context.

Tier 1: Immediate Containment in Coastal Urban Agglomerations. The southeastern coastal provinces (e.g., Guangdong, Zhejiang), which contain 32.2% of the national core invasion area, should be the focus of immediate, aggressive intervention. Management must emphasize “source control”: strengthening quarantine at major ports (e.g., Shanghai, Shenzhen) and implementing “zero-tolerance” eradication programs for nascent populations using rapid response teams.

Tier 2: Resilience Building within and around Protected Areas. For the core habitat already within PAs, the priority shifts to internal resilience enhancement. PA managers should use our maps to target native vegetation restoration and create invasion-resistant buffer zones.

Tier 3: Transportation infrastructure, particularly highways, was identified as a dominant dispersal pathway. Integrating invasive species control into the maintenance schedules of transport ministries is crucial. Re-vegetation of road and railway verges with native species can disrupt the linear spread of IAPs. This species- and site-specific approach enables a shift from costly, blanket control measures to cost-effective, precision management.

Furthermore, our species-specific suitability analysis allows for differentiated species management. The 13 species classified as highly suitable (e.g., *Aemella oleracea*, HSI = 0.916) with broad environmental tolerance should be national priorities for eradication and monitoring. This nuanced, species-and-site-specific approach enables a shift from costly, blanket control measures to cost-effective, precision management.

Our results, consistent with previous research, indicate that transportation infrastructure shows a stronger statistical association with IAPs spread than natural water systems, suggesting that these features may be important pathways for dispersal. The mean risk values for the three pathways—highways (0.00548), railways (0.00461), and rivers (0.00327)—exhibit a clear gradient. The average risk value for highways is 67.7% higher than that for rivers, consistent with a positive correlation between human activity intensity and invasion risk. Although riparian areas experience strong invasion pressures worldwide [22], rivers exhibit a lower average risk value; however, their linear connectivity and extensive networks result in a large potential impact area [23]. Notably, river corridors contain the largest contiguous hotspot area (146 × 10^4^ km^2^), followed by highways (127 × 10^4^ km^2^) and railways (109 × 10^4^ km^2^). The combined hotspot area covers 269 × 10^4^ km^2^, suggesting substantial cumulative spatial extent and potential for interactions among multiple dispersal pathways. Dominance analysis shows that highways are the dominant pathway in 94.94% of the study area, suggesting that the high connectivity of road networks may contribute to efficient dispersal corridors for invasive species. The geo-detector analysis further confirmed significant interactive effects between highways, railways, and nighttime light intensity (DMSP; q = 0.562). The highest dispersal risks were identified in major urban agglomerations with dense transportation networks, such as the Beijing-Tianjin-Hebei region, the Pearl River Delta, and the Yangtze River Delta.

Based on our spatial analysis, we propose a climate-smart adaptive Bio-security framework [24]. Urgent intervention should focus on the core habitat gap (17.85 × 10^4^ km^2^). The top 20 county-level administrative units account for approximately 13.5 × 10^4^ km^2^ of this gap, exhibiting significant spatial aggregation. These prioritized counties are primarily concentrated in three major economic zones: the Pearl River Delta (e.g., Dongguan, Zhongshan, Nanhai; 8 counties), the Yangtze River Delta (e.g., Pudong New Area, Jiangning District, Kunshan; 5 counties), and the Beijing-Tianjin-Hebei region (e.g., Daxing District, Tongzhou District, Chang’an District; 3 counties).

### 3.4. Theoretical Integration and Future Directions

For alien species to establish successfully in a recipient community, they must possess functional traits that confer competitive superiority over native species or allow them to exploit unoccupied niche space [25], such as extended flowering periods that enhance pollinator attraction [26].

Our findings at the national scale align with several foundational theories in invasion ecology. The “two-high-one-low” pattern (high DMSP, high Bio12, low Bio2) aligns with predictions from the “fluctuating resources” theory, as it identifies landscapes where resource availability (water, nutrients in disturbed soils) is predictably high and Biotic stress (temperature variability) is low, which may represent windows of opportunity for invasion. Simultaneously, it reflects the “empty niche” hypothesis, as intense human disturbance simplifies native communities, vacating niches that generalist IAPs readily fill.

This study has several limitations and future research needs. While our integrated framework mitigates over-prediction, the reliance on climatic baselines (1970–2000) may not fully capture recent warming trends. Future work should model invasion risk under SSP-RCP climate scenarios to anticipate shifting hotspots. Furthermore, the spatial patterns are driven by Biotic and anthropogenic factors; incorporating biotic interactions (e.g., competition, facilitation) through models like HMSC could refine predictions. Finally, the InVEST model parameters, though calibrated, involve subjectivity; a comprehensive sensitivity analysis of threat weights and decay functions would strengthen the habitat degradation assessment.

By integrating species distribution modeling with habitat quality assessment, this study identifies areas where environmental suitability for invasion coincides with elevated ecosystem vulnerability. The analysis of factor interactions, identification of protection gaps, and proposal of a tiered management framework offer an evidence-based pathway to support China’s transition from reactive to proactive invasive species management.

For Tier 1 Priority Zones—the southeastern coastal urban agglomerations—we recommend implementing an integrated “source control, monitoring, and rapid response” system. These include: (i) strengthening quarantine inspections at ports, airports, and major highway interchanges; (ii) deploying a real-time surveillance network that combines citizen science with drone-based remote sensing; and (iii) establishing specialized rapid-response teams to enforce zero-tolerance eradication of nascent populations, effectively reducing the risk of establishment (Supplementary Figure S1).

Tier 2 Priority Zones (Core Invasion Gaps within Protected Areas): A combined strategy of ecological restoration and buffer isolation is recommended. Key actions include: (i) implementing native species-based ecological restoration projects to improve habitat quality and landscape connectivity, thereby enhancing ecosystem resilience; and (ii) establishing invasion-control buffer zones along the boundaries between protected areas, urban lands, and farmlands, with regular clearing of invasive species to block dispersal pathways into core natural reserves.

Transportation Corridors: Linear infrastructure (e.g., highways and railways) should be formally incorporated into invasive plant management plans. Recommended practices include: (i) utilizing native plant species in slope stabilization and greening projects, while prohibiting species with invasive traits; and (ii) instituting regular inspection and clearing regimes to disrupt their function as invasion conduits.

### 3.5. Study Limitations

Although the integrated MaxEnt-InVEST approach was employed to mitigate over-prediction by MaxEnt and to identify overlapping hotspots of biodiversity and habitat quality-thus providing a more robust assessment of invasion-related ecosystem impacts [27], this study has several limitations. First, despite using the highest resolution county-level invasive plant database currently available, we retained only species with more than five occurrence records (*n* = 293) to ensure model stability. This exclusion of species with sparse records (from over 660 documented invasive species in China) may introduce sampling bias and under-represent recently introduced species. Moreover, cumulative richness maps derived from aggregating individual SDMs propagate uncertainties from each species-level model, including threshold selection and sampling bias. The core invasion habitats identified here should therefore be interpreted as hypothesis-generating priority areas requiring field validation rather than definitive invasion fronts.

Second, the climatic variables used in this study (WorldClim, 1970–2000) represent the most recent long-term climate baselines available at the time of analysis. While these data provide a stable reference for characterizing species’ fundamental climatic niches, we acknowledge that future updates to WorldClim could further refine the accuracy of habitat suitability predictions [14]. Future studies should incorporate updated climate data as they become available, as well as future climate scenarios (e.g., SSP-RCP pathways), to assess how climate change may alter invasion risk patterns.

Third, key parameters in the InVEST model, though calibrated, involve a degree of subjectivity derived from literature and expert knowledge. The selection of threat weights and sensitivity scores may influence the delineation of core invasion habitats. A comprehensive sensitivity analysis of these parameters would enhance the robustness of the results and should be prioritized in future research.

Fourth, the spatial patterns of IAPs are influenced by multiple biotic and abiotic factors. Although this study incorporated key anthropogenic factors such as road networks and nighttime lights alongside climatic variables, it did not simulate temporal dispersal dynamics or species interactions. Future work should adopt integrated model frameworks such as Hierarchical Model of Species Communities (HMSC) to quantify facilitative and competitive interactions among species [28,29,30], and should incorporate multi-temporal remote sensing data and future climate scenarios (e.g., SSP-RCP pathways) to better capture the spatiotemporal dynamics of plant invasions in China.

## 4. Conclusions

This study applied an integrated MaxEnt-InVEST modeling framework to assess habitat suitability and quality for 293 invasive plant species across China. Our findings highlight three principal insights: (1) Regions with high nighttime light intensity (DMSP), high annual precipitation (Bio12), and low diurnal temperature range (Bio2) exhibit the highest invasion risk and may warrant stringent monitoring. (2) High-intensity disturbance zones are predicted to be the most susceptible and vulnerable habitats for invasive plants in China. (3) A spatial mismatch was identified, with 88.82% of core invasion habitats lying outside the current protected area network, primarily in southeastern coastal urban agglomerations.

In conclusion, this study provides a national-scale, spatially explicit assessment of invasion risk for 293 plant species in China, integrating species distribution modeling, habitat quality assessment, and protected area gap analysis. The maps of core invasion habitats and priority control areas can guide resource allocation for patrol and eradication, enabling a shift from broad, reactive measures to precision management. The methodological framework developed here is transferable to other countries and regions facing similar challenges from biological invasions, particularly where high-resolution occurrence data and environmental layers are available. The finding that core invasion habitats are concentrated outside protected areas in human-dominated landscapes highlights the need for conservation strategies that extend beyond traditional reserve boundaries-a lesson with global relevance as signatories to the Kunming-Montreal Global Biodiversity Framework work toward Target 6 on invasive species management.

## 5. Materials and Methods

### 5.1. Species Distribution Data

**Figure 9:**
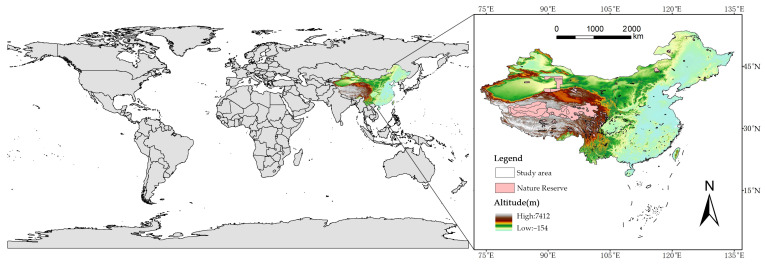
Location of study area and distribution of protected areas.

This study utilized a comprehensive, county-level distribution database of invasive alien plants in China, which initially contained occurrence records for 400 species across 2684 counties [31]. The data were compiled from multiple authoritative sources, including the Chinese Virtual Herbarium (CVH) and the Global Biodiversity Information Facility (GBIF), and regional Biodiversity databases, and underwent rigorous taxonomic verification and coordinate validation. To minimize spatial autocorrelation and sampling bias, we implemented a two-step data screening process. First, a spatial filter was applied to retain only one occurrence record per species within each 5 × 5 km grid cell, and species with fewer than five unique occurrence records were excluded for subsequent analysis [15]. This process resulted in a final dataset comprising 293 invasive plant species, with a total of 28,979 occurrence points (Figure 9). The final dataset represents those with sufficient occurrence data for reliable modeling (Table S1), rather than a complete list of all invasive plants documented in China (over 660 species). Secondly, to account for heterogeneous sampling effort across the study region, we selected background points using a bias file derived from the density of all occurrence records, following the target-group background approach. This method weights background sampling according to the spatial distribution of overall recording intensity, thus alleviating the influence of spatially clustered sampling on model predictions.

### 5.2. Environmental Variables

Based on ecological relevance and findings from prior studies on plant invasions in China, we initially selected 25 environmental variables across three categories: climate, habitat conditions, and anthropogenic disturbance [32,33]. Nineteen bioclimatic variables for the period 1970–2000 were obtained from the WorldClim database (v2.2), which represents the most recent long-term climate baselines available for species distribution modeling. Given that both the maximum temperature of the warmest month and the minimum temperature of the coldest month are highly correlated with annual mean temperature (Bio1), as are the mean temperatures of the wettest and driest quarters, we retained annual mean temperature (Bio1) as a representative metric to reduce multicollinearity. Similarly, since annual temperature range is derived from extreme monthly temperatures and isothermality is calculated from diurnal and annual ranges, we selected mean diurnal range (Bio2) and temperature seasonality (Bio4) for analysis. To capture variability in precipitation, we noted that precipitation of the wettest month and warmest quarter is highly correlated with annual precipitation (Bio12); hence, Bio12 was selected as a key variable. Precipitation of the driest month was excluded due to its conceptual overlap with precipitation of the driest quarter.

To address multicollinearity among candidate predictors, we calculated pearson correlation coefficients and variance inflation factors (VIF). Variables with |r| > 0.8 or VIF > 5 were considered collinear and sequentially removed, prioritizing retention of ecologically relevant predictors. The final climatic variables retained for the modeling were: Bio1 (annual mean temperature, °C), Bio2 (mean diurnal range, °C), Bio4 (temperature seasonality, °C × 100), Bio12 (annual precipitation, mm), and Bio15 (precipitation seasonality, coefficient of variation). The final set of 11 variables (Table 1) had VIF values < 5, confirming the absence of severe multicollinearity. A correlation heat-map is shown in Supplementary Figure S2.

To represent anthropogenic pressures, DMSP was generated from annual Composed NPP-VIIRS NTL data (2021–2023) [34]. While there is a temporal offset between the climate and anthropogenic data, this approach is standard practice in invasion risk mapping: climate baselines define where species could potentially establish, while recent anthropogenic data indicate where human-mediated introduction and disturbance currently facilitate invasions. These data were then averaged, normalized, and log-transformed to reduce the influence of extreme values. Global land cover and cropland percentage data were obtained from the ESA GloCover 2009 project. These data were reclassified into seven land-use/land-cover (LULC): high- intensity disturbance zones, degraded forestland, shrubland, wetland invasion hotspots, degraded grassland, transportation corridors, and primary vegetation areas. Road density (RD) was calculated using OpenStreetMap (OSM) data. All spatial datasets were harmonized to the WGS84 geographic coordinate system (EPSG:4326). The normalized difference vegetation index (NDVI) was derived from NASA MOD13A3 data for 2024 data using the maximum value composite (MVC) method and was subsequently converted to fractional vegetation cover (FVC). Soil pH (0–5 cm depth) was obtained from the SoilGrids database. Data gaps were filled using a random forest model that established nonlinear relationships between pH and other environmental variables. All raster layers were resampled to a unified 1 km resolution and clipped to the national boundary of China using ArcGIS 10.8 (Table 1). Finally, the 11 environmental variables were converted to ASCII format using Raster to ASCII tool in ArcGIS [35].

### 5.3. Analysis

#### 5.3.1. Habitat Suitability Modeling with MaxEnt

The Maximum Entropy (MaxEnt) model (v 3.4.1) was employed to predict the habitat suitability for each of the 293 IAPs. Species occurrence data and the 10 environmental layers were imported into the model. For each species run, 75% of the occurrence points were randomly selected for model training, and the remaining 25% were used for validation [36]. Model settings included 10 bootstrap replicates, 10,000 background points, and a maximum of 500 iterations [37]. The jackknife test was used to evaluate the relative contribution of each environmental variable [38]. The model output is a logistic raster (0–1) where each pixel value represents the Habitat Suitability Index (HSI), interpreted as the probability of species presence [39].Continuous HSI outputs were converted to binary maps using the maximum training sensitivity plus specificity threshold, which balances omission and commission errors and performs well across species with varying prevalence. Model performance was evaluated using the Area under the Receiver Operating Characteristic Curve (AUC). The evaluation criteria were: 0.5–0.7 (poor), 0.7–0.8 (fair), 0.8–0.9 (good), and 0.9–1.0 (excellent) [40]. The AUC values, along with the training sensitivity and specificity thresholds for all 293 species, are provided in Supplementary Table S2.

#### 5.3.2. Habitat Quality Assessment with the InVEST-HQ Module

The Integrated Valuation of Ecosystem Services and Trade (InVEST) model is designed to assess key ecosystem services, supporting ecological management and decision-making through the evaluation of habitat quality [41].Habitat quality and degradation were assessed using the Habitat Quality module of the InVEST model (v.3.16.1).The model evaluates habitat quality based on land use/cover types, the intensity and spatial influence of threat factors, and the sensitivity of each habitat type to those threats [42].The computation is based on habitat suitability values, the intensity of various threats, and the sensitivity of each habitat type to those threats [43]. The model was calibrated using an iteratively optimized K parameter (initial K = 0.05, optimized K = 0.434).

With reference to previous studies that have established methodologies for threat factors in the InVEST model [22,44,45], seven sources of anthropogenic pressure were identified as threats: highways, railways, urban areas, ports, farmland, rivers, and tourism areas. The weight, maximum influence distance, and decay type (linear or exponential) for each threat factor were determined based on literature review and the specific context of China (Table 2). To ensure the parameters reflect both established research and the specific context of the study area, the threat weights, maximum impact distances, and decay types were determined through a systematic review of published InVEST applications in China (*n* = 23 studies) and consultation with three independent experts in invasion ecology and conservation planning [22]. The sensitivity of each land cover type (e.g., forest, grassland, wetland, cropland) to each threat factor was assigned a value between 0 (no sensitivity) and 1 (highest sensitivity), referencing existing studies and expert knowledge [45]. The model outputs a habitat quality index raster (0–1), where values closer to 1 indicate higher habitat quality and lower degradation, and values closer to 0 indicate poorer quality and higher degradation pressure [46].

#### 5.3.3. Analysis of Driving Factors Using the Geo-Detector Model

To quantify the influence of individual environmental factors and their interactions on the spatial pattern of IAP species richness, we employed the geo-detector model. Geographic detectors are a set of statistical methods for detecting spatial heterogeneity and identifying its driving factors [47]. To ensure spatial representativeness and uniformity of samples, we generated 50,000 random points across mainland China using the “Create Random Points”. A species richness raster (Y-Richness) was created by summing the predicted distribution layers of all 293 invasive plant species derived from the MaxEnt model, where each pixel value represents the predicted species at a given location. The independent variables consisted of the previously selected environmental factors [48]. To meet the data requirements of the geo-detector model, continuous variables were reclassified into five categories using the Jenks natural breaks method, with the exception of soil PH. Given its clear ecological implications, soil pH was classified manually. All geo-detector analyses were performed in R 4.5.2 using the “geo-detector” package. We executed the four core modules of the model in sequence: factor detector (q-statistic), interaction detector, risk detector, and ecological detector. These analyses aimed to: (1) quantify the independent explanatory power of each factor on the spatial heterogeneity of invasion risk; (2) assess interactions between factor pairs; (3) identify risk zones based on factor values; and (4) statistically compare the spatial differentiation effects of different factors.

#### 5.3.4. Model Integration and Analysis

The conceptual basis of this study rests on the hypothesis that the core invasion habitats must simultaneously satisfy two conditions: (1) high environmental suitability for the establishment and reproduction of invasive plants, and (2) significant exposure to anthropogenic disturbance and ecological pressure, which degrades native ecosystem resilience. Individually, the MaxEnt model predicts where invasive species could potentially establish based on environmental niches, while the InVEST model identifies where native habitats are most vulnerable due to human pressures [49]. Their integration targets the intersection of these two dimensions—areas where the probability of invasion success and the potential for severe ecological impact are concurrently highest. This approach excludes regions that are either unsuitable for invasion or, despite being suitable, retain robust native communities capable of resistance, thereby directing management attention to the most consequential areas. The integration procedure was conducted as follows:

Habitat Suitability Zone: Binary layers for all 293 species generated by MaxEnt were summed in ArcGIS to create a potential species richness raster [50]. This stacking approach implicitly assumes equal weight for each species. The raster was classified into five levels (high, relatively high, medium, relatively low, low) using the Jenks natural breaks method [51]. Areas classified with high, relatively high, and medium richness were categorized as “High Suitability Habitats”.

Habitat Quality/Degradation Zone: The habitat quality index raster output from the InVEST model was similarly classified into five levels using the Jenks method [52]. In this context, lower habitat quality values indicate higher degradation pressure and ecosystem vulnerability. Consequently, areas with medium quality and below (i.e., higher degradation) were categorized as “High-Vulnerability Habitats.”

Identification of Core Invasion Habitats: The “Core Invasion Habitat” was defined as the spatial intersection of the “High Suitability Habitats” (from MaxEnt) and “High-Vulnerability Habitats” (from InVEST). Areas identified by only one model were assigned to a secondary priority category [15].

Conservation Gap Analysis: The spatial boundaries of China’s National Nature Reserves were overlaid with the “Core Invasion Habitat” layer [53]. The area of core habitat located outside the protected area network was calculated and defined as the “Conservation Gap,” pinpointing regions of high invasion risk that currently lack formal protection.

## Figures and Tables

**Table 1:** Enviromental factors used for analysis of invasive habitats in China by MaxEnt model.

Factors Type	Variable Abbreviation	Description
Climate factors	Bio1	Annual MeanTemperature
	Bio2	MeanDiurnalRange
	Bio4	Temperature Seasonality
	Bio12	Annual Precipitation
	Bio15	Precipitation Seasonality
Habitat factors	SoilPH	SoilhydrogenIon Concentration
	NDVI	Normalized difference vegetation index
	CP	Cropland Percentage
Human disturbance factors	DMSP	Nighttime lighting
	RD	Road Density

**Table 2:** Maximum impact distance and weight over each threat affecting habitat quality.

Threat Factor	Maximum Impact Distance (km)	Weight	Decay Type
Highway	2	0.85	linear
Railway	1	0.65	linear
Urban area	4	0.9	linear
Port	1.5	0.95	exponential
Farmland	8	0.7	linear
River	0.5	0.5	exponential
Tourism	3.5	0.65	exponential

## Data Availability

The data and code that support the findings of this study are openly available in Zenodo at https://doi.org/10.5281/zenodo.18320432. Species occurrence data were obtained from the comprehensive county-level distribution database of alien and invasive plants in China at https://doi.org/10.1002/ecy.70084. Climate and elevation data were obtained from WorldClim 2.1 at https://www.worldclim.org (accessed 21 November 2025). Nighttime lighting was obtained from an extended time series (2000–2018) of global NPP-VIIRS-like nighttime light data from a cross-sensor calibration at https://doi.org/10.5194/essd-13-889-2021. Land cover data were obtained from the Glo-Cover 2009 at https://due.esrin.esa.int/page_globcover.php (accessed 22 November 2025). NDVI data were obtained from the NASA MOD13A3 at https://www.earthdata.nasa.gov (accessed 22 November 2025). Soil pH was obtained from the Global Soil pH Data at https://soilgrids.org (accessed 23 November 2025). No specific permits were required for plant occurrence data collection. The original contributions presented in the study are included in the article; further inquiries can be directed to the corresponding authors.

## Supplementary Materials

The following are available online at https://www.mdpi.com/article/10.3390/plants15060898/s1, Table S1: Plant invasion levels and distribution points statistic in China, Table S2: The AUC and the equal training sensitivity and specificity logistic threshold of MaxEnt models, Figure S1: Provincial and county-level distribution of high-risk invasion zones in China, Figure S2: Interaction heatmap of environmental factors.
