# Supplementary material for: National-Scale Conservation Gaps and Priority Areas for Invasive Plant Control in China: An Integrated MaxEnt-InVEST Framework
Source: Plants (Basel). 2026 Mar 13;15(6):898. doi: 10.3390/plants15060898 (PMC13030478; doi:10.3390/plants15060898)
Supplement: Supplementary file 1 [file plants-15-00898-s001.zip › plants-4165540-supplementary.pdf]

Supplementary tables

Table S1 Plant invasion levels and distribution points statistic in China.

Family	Genus	Species	Invasion level	Number of distribution points
Alismataceae	Limnocharis	*Limnocharis flava*	4	13
Amaranthaceae	Alternanthera	*Alternanthera paronychioides*	3	24
		Alternanthera philoxeroides	3	248
		Alternanthera pungens	4	10
Amaranthaceae	Amaranthus	*Amaranthus albus*	3	14
		Amaranthus blitoides	3	8
		Amaranthus blitum	3	92
		Amaranthus dubius	4	5
		Amaranthus hybridus	4	87
		Amaranthus palmeri	5	5
		Amaranthus retroflexus	4	75
		Amaranthus spinosus	4	100
		Amaranthus viridis	4	256
Amaranthaceae	Chenopodiastrum	*Chenopodium hybridum*	3	64
	Dysphania	*Dysphania ambrosioides*	4	90
	Gomphrena	*Gomphrena celosioides*	4	117
Amaryllidaceae	Zephyranthes	*Zephyranthes carinata*	2	15
Anacardiaceae	Rhus	*Rhus typhina*	3	26
Apiaceae	Cyclospermum	*Cyclospermum leptophyllum*	3	30
	Daucus	*Daucus carota*	2	145
	Eryngium	*Eryngium foetidum*	2	48
Apocynaceae	Asclepias	*Asclepias curassavica*	2	161
	Catharanthus	*Catharanthus roseus*	2	228
Araceae	Pistia	*Pistia stratiotes*	5	150
Araliaceae	Hydrocotyle	*Hydrocotyle verticillata*	3	182
Asparagaceae	Agave	*Agave americana*	3	9
Asteraceae	Acmella	*Acmella ciliata*	2	6
		Acmella oleracea	2	10
		Acmella uliginosa	2	70
		Ageratina adenophora	2	24
	Ageratina	*Ageratina riparia*	5	13
	Ageratum	*Ageratum conyzoides*	5	390
		Ageratum houstonianum	5	395
	Ambrosia	*Ambrosia artemisiifolia*	5	55
		Ambrosia trifida	5	11
	Austroeupatorium	*Austroeupatorium inulifolium*	4	17
	Bidens	*Bidens alba*	4	574

	Bidens bipinnata	4	78
	Bidens frondosa	3	23
	Bidens pilosa	5	497
Calyptocarpus	*Calyptocarpus vialis*	3	115
Centaurea	*Centaurea cyanus*	2	47
Chromolaena	*Chromolaena odorata*	5	222
Cichorium	*Cichorium intybus*	3	20
Coreopsis	*Coreopsis lanceolata*	2	16
	Coreopsis tinctoria	2	8
Cosmos	*Cosmos bipinnatus*	2	123
	Cosmos sulphureus	2	52
Crassocephalum	*Crassocephalum crepidioides*	4	435
	Crassocephalum rubens	3	9
Crepis	*Crepis tectorum*	3	12
Elephantopus	*Elephantopus tomentosus*	3	20
Emilia	*Emilia fosbergii*	3	22
	Emilia praetermissa	2	110
Erechtites	*Erechtites hieraciifolius*	3	33
	Erechtites valerianifolius	3	137
Erigeron	*Erigeron annuus*	4	178
	Erigeron bellioides	3	35
	Erigeron bonariensis	4	140
	Erigeron canadensis	4	287
	Erigeron philadelphicus	3	31
	Erigeron sumatrensis	4	297
Gaillardia	*Gaillardia pulchella*	2	33
Galinsoga	*Galinsoga parviflora*	4	93
	Galinsoga quadriradiata	4	204
Gamochaeta	*Gamochaeta pensylvanica*	3	129
	Gamochaeta purpurea	3	70
Gymnocoronis	*Gymnocoronis spilanthoides*	3	36
Helianthus	*Helianthus tuberosus*	3	46
Hypochaeris	*Hypochaeris albiflora*	3	12
	Hypochaeris radicata	3	17
Lactuca	*Lactuca serriola*	3	23
Leucanthemum	*Leucanthemum vulgare*	3	22
Mikania	*Mikania micrantha*	5	409
Parthenium	*Parthenium hysterophorus*	5	183
Pluchea	*Pluchea carolinensis*	3	120
	Pluchea sagittalis	3	91
Praxelis	*Praxelis clematidea*	4	226
Pseudelephantopus	*Pseudelephantopus spicatus*	3	27
Senecio	*Senecio vulgaris*	3	38

	Silphium	*Silphium perfoliatum*	2	5
	Silybum	*Silybum marianum*	3	9
	Solidago	*Solidago canadensis*	5	43
	Soliva	*Soliva anthemifolia*	3	141
		Soliva sessilis	3	65
	Sonchus	*Sonchus asper*	3	188
	Sphagneticola	*Sphagneticola trilobata*	5	275
	Symphyotrichum	*Symphyotrichum subulatum*	4	244
	Synedrella	*Synedrella nodiflora*	3	151
	Tagetes	*Tagetes erecta*	1	26
	Taraxacum	*Taraxacum officinale*	3	110
	Tarlmounia	*Tarlmounia elliptica*	1	7
	Tithonia	*Tithonia diversifolia*	4	158
	Tragopogon	*Tragopogon dubius*	3	14
	Tridax	*Tridax procumbens*	3	292
	Zinnia	*Zinnia peruviana*	2	10
Basellaceae	Anredera	*Anredera cordifolia*	5	259
Begoniaceae	Begonia	*Begonia cucullata*	2	22
Boraginaceae	Symphytum	*Symphytum officinale*	2	20
Brassicaceae	Lepidium	*Coronopus didymus*	3	20
		Lepidium densiflorum	3	13
		Lepidium virginicum	3	174
	Nasturtium	*Nasturtium officinale*	2	64
Cabombaceae	Cabomba	*Cabomba caroliniana*	5	10
Campanulaceae	Hippobroma	*Hippobroma longiflora*	3	38
Cannabaceae	Cannabis	*Cannabis sativa*	1	140
Caryophyllaceae	Agrostemma	*Agrostemma githago*	3	7
	Cerastium	*Cerastium glomeratum*	3	99
Cleomaceae	Sieruela	*Cleome rutidosperma*	3	274
Commelinaceae	Callisia	*Callisia repens*	3	62
	Tradescantia	*Tradescantia fluminensis*	4	65
		Tradescantia zebrina	4	139
Convolvulaceae	Cuscuta	*Cuscuta campestris*	4	137
	Evolvulus	*Evolvulus nummularius*	2	49
	Ipomoea	*Ipomoea alba*	3	20
		Ipomoea cairica	5	384
		Ipomoea coccinea	3	5
		Ipomoea hederacea	3	32
		Ipomoea indica	5	219
		Ipomoea lacunosa	3	9
		Ipomoea mauritiana	3	12
		Ipomoea nil	3	193
		Ipomoea purpurea	3	127

		Ipomoea quamoclit	3	69
		Ipomoea triloba	3	291
Convolvulaceae	Jacquemontia	*Jacquemontia tamnifolia*	3	9
Crassulaceae	Kalanchoe	*Bryophyllum pinnatum*	3	7
Cucurbitaceae	Melothria	*Melothria pendula*	3	180
Cyperaceae	Cyperus	*Cyperus esculentus*	3	5
		Cyperus surinamensis	3	33
		Kyllinga polyphylla	3	40
Euphorbiaceae	Croton	*Croton bonplandianus*	3	5
	Euphorbia	*Euphorbia dentata*	3	5
		Euphorbia heterophylla	3	136
		Euphorbia hirta	3	458
		Euphorbia hypericifolia	3	166
		Euphorbia maculata	3	98
		Euphorbia marginata	2	6
		Euphorbia nutans	3	12
		Euphorbia peplus	3	13
		Euphorbia prostrata	3	235
		Euphorbia pulcherrima	1	27
		Euphorbia serpens	3	131
		Euphorbia tirucalli	3	19
Euphorbiaceae	Ricinus	*Ricinus communis*	4	348
Fabaceae	Acacia	*Acacia dealbata*	4	11
		Acacia mearnsii	4	8
	Aeschynomene	*Aeschynomene americana*	2	66
	Albizia	*Albizia lebbeck*	3	34
	Cajanus	*Cajanus cajan*	1	133
	Calopogonium	*Calopogonium mucunoides*	3	28
	Centrosema	*Centrosema pubescens*	2	135
	Chamaecrista	*Chamaecrista mimosoides*	3	39
	Clitoria	*Clitoria ternatea*	2	144
	Coronilla	*Coronilla varia*	3	10
	Crotalaria	*Crotalaria micans*	2	29
		Crotalaria pallida	3	130
		Crotalaria trichotoma	3	105
	Desmodium	*Desmodium tortuosum*	3	66
	Indigofera	*Indigofera suffruticosa*	3	65
	Leucaena	*Leucaena leucocephala*	5	368
	Macroptilium	*Macroptilium atropurpureum*	2	195
		Macroptilium lathyroides	2	95
	Medicago	*Medicago polymorpha*	3	44
		Medicago sativa	1	189
	Melilotus	*Melilotus albus*	3	56

		Melilotus indicus	3	49
		Melilotus officinalis	3	124
	Mimosa	*Mimosa bimucronata*	4	17
		Mimosa diplotricha	5	139
		Mimosa pigra	5	31
		Mimosa pudica	4	339
	Robinia	*Robinia pseudoacacia*	4	83
	Senna	*Senna alata*	3	128
		Senna bicapsularis	3	32
		Senna hirsuta	3	13
		Senna occidentalis	3	143
		Senna sophera	3	16
	Sesbania	*Sesbania bispinosa*	2	8
		Sesbania cannabina	1	226
	Stylosanthes	*Stylosanthes guianensis*	2	10
	Tephrosia	*Tephrosia candida*	2	18
	Trifolium	*Trifolium hybridum*	2	9
		Trifolium pratense	2	74
		Trifolium repens	3	311
	Vicia	*Vicia villosa*	2	22
Geraniaceae	Geranium	*Geranium carolinianum*	3	92
Haloragaceae	Myriophyllum	*Myriophyllum aquaticum*	5	132
Hydrocharitaceae	Elodea	*Egeria densa*	5	64
Iridaceae	Crocosmia	*Crocosmia crocosmiiflora*	2	39
	Iris	*Iris pseudacorus*	4	46
Lamiaceae	Hyptis	*Hyptis brevipes*	3	21
		Hyptis rhomboidea	3	69
		Hyptis suaveolens	2	29
	Salvia	*Salvia coccinea*	2	15
	Stachys	*Stachys arvensis*	3	31
Linderniaceae	Lindernia	*Lindernia rotundifolia*	2	44
Lythraceae	Ammannia	*Ammannia coccinea*	3	16
	Cuphea	*Cuphea carthagenensis*	3	192
	Sonneratia	*Sonneratia apetala*	3	10
Malvaceae	Abutilon	*Abutilon theophrasti*	4	140
	Corchorus	*Corchorus olitorius*	2	25
	Herissantia	*Herissantia crispa*	3	12
	Hibiscus	*Hibiscus trionum*	3	103
	Malvastrum	*Malvastrum coromandelianum*	3	212
	Sida	*Sida acuta*	4	79
	Waltheria	*Waltheria indica*	3	51
Marantaceae	Thalia	*Thalia dealbata*	3	36
Myrtaceae	Eucalyptus	*Eucalyptus robusta*	4	39

Nyctaginaceae	Mirabilis	*Mirabilis jalapa*	2	214
Onagraceae	Ludwigia	*Ludwigia decurrens*	4	39
	Oenothera	*Gaura parviflora*	3	6
		Oenothera biennis	3	28
		Oenothera drummondii	3	10
		Oenothera glazioviana	3	13
		Oenothera laciniata	3	108
		Oenothera rosea	3	25
		Oenothera speciosa	3	60
		Oenothera tetraptera	2	9
Oxalidaceae	Oxalis	*Oxalis articulata*	3	46
		Oxalis triangularis	2	29
Papaveraceae	Argemone	*Argemone mexicana*	4	27
Passifloraceae	Passiflora	*Passiflora foetida*	3	34
		Passiflora suberosa	3	334
Phyllanthaceae	Phyllanthus	*Phyllanthus tenellus*	2	255
Phytolaccaceae	Phytolacca	*Phytolacca americana*	4	313
	Rivina	*Rivina humilis*	2	171
Piperaceae	Peperomia	*Peperomia pellucida*	2	71
Plantaginaceae	Mecardonia	*Mecardonia procumbens*	3	137
	Plantago	*Plantago aristata*	3	7
		Plantago virginica	3	73
	Stemodia	*Stemodia verticillata*	2	60
	Veronica	*Veronica arvensis*	3	51
		Veronica hederifolia	3	5
		Veronica persica	4	242
		Veronica polita	3	55
Poaceae	Aegilops	*Aegilops tauschii*	1	15
	Avena	*Avena fatua*	4	88
	Axonopus	*Axonopus compressus*	3	140
	Bromus	*Bromus catharticus*	3	49
	Cenchrus	*Cenchrus echinatus*	4	167
		Pennisetum purpureum	3	127
	Hordeum	*Hordeum jubatum*	2	10
	Lolium	*Lolium multiflorum*	3	32
		Lolium perenne	2	26
		Lolium temulentum	4	7
	Melinis	*Melinis repens*	4	252
	Panicum	*Panicum repens*	4	102
	Paspalum	*Paspalum conjugatum*	3	203
		Paspalum dilatatum	3	48
		Paspalum distichum	3	50
		Paspalum urvillei	3	126

	Setaria	*Pennisetum polystachion*	4	44
	Sorghum	*Sorghum halepense*	5	72
	Urochloa	*Brachiaria mutica*	3	76
Polygalaceae	Polygala	*Polygala paniculata*	2	59
Polygonaceae	Antigonon	*Antigonon leptopus*	4	77
Pontederiaceae	Pontederia	*Eichhornia crassipes*	3	35
Portulacaceae	Portulaca	*Portulaca pilosa*	3	177
Ranunculaceae	Ranunculus	*Ranunculus muricatus*	3	22
Rubiaceae	Mitracarpus	*Mitracarpus hirtus*	3	10
	Richardia	*Richardia brasiliensis*	3	31
		Richardia scabra	3	39
Rubiaceae	Spermacoce	*Spermacoce remota*	3	129
Salviniaceae	Azolla	*Azolla filiculoides*	4	22
	Salvinia	*Salvinia molesta*	5	44
Solanaceae	Datura	*Datura innoxia*	4	13
		Datura metel	4	43
		Datura stramonium	4	192
	Nicandra	*Nicandra physalodes*	3	60
	Physalis	*Physalis angulata*	3	297
		Physalis peruviana	3	56
		Physalis pubescens	3	53
	Solanum	*Solanum americanum*	4	438
		Solanum capsicoides	4	147
		Solanum diphyllum	3	248
		Solanum erianthum	4	186
		Solanum mauritianum	4	9
		Solanum pseudocapsicum	3	160
		Solanum scabrum	3	30
		Solanum sisymbriifolium	3	271
		Solanum torvum	5	74
		Solanum viarum	4	353
Talinaceae	Talinum	*Talinum fruticosum*	2	38
		Talinum paniculatum	2	262
Urticaceae	Pilea	*Pilea microphylla*	3	384
Verbenaceae	Lantana	*Lantana camara*	5	395
		Lantana montevidensis	3	32
	Stachytarpheta	*Stachytarpheta cayennensis*	3	13
		Stachytarpheta jamaicensis	3	54
	Verbena	*Verbena bonariensis*	3	44
		Verbena brasiliensis	3	17
Vitaceae	Parthenocissus	*Parthenocissus quinquefolia*	2	37

3 Note: 1.Blank cells indicate the same Family or Genus as the preceding row. Only species with ≥5 distribution points are included.

Table S2 The AUC and the equal training sensitivity and specificity logistic threshold of MaxEnt models.

Species	AUC	Threshold	Species	AUC	Threshold	Species	AUC	Threshold
Abutilon Theophrasti	0.951	0.027	*Erigeron Bonariensis*	0.972	0.012	*Parthenium Hysterophorus*	0.989	0.008
Acacia Dealbata	0.927	0.103	*Erigeron Canadensis*	0.948	0.023	*Parthenocissus Quinquefolia*	0.968	0.016
Acacia Mearnsii	0.932	0.106	*Erigeron Philadelphicus*	0.984	0.005	*Paspalum Conjugatum*	0.992	0.007
Acmella Ciliata	0.987	0.009	*Erigeron Sumatrensis*	0.986	0.007	*Paspalum Dilatatum*	0.992	0.007
Acmella Oleracea	0.999	0.003	*Eryngium Foetidum*	0.982	0.009	*Paspalum Distichum*	0.983	0.01
Acmella Uliginosa	0.997	0.004	*Eucalyptus Robusta*	0.984	0.013	*Paspalum Urvillei*	0.993	0.005
Aegilops Tauschii	0.904	0.063	*Euphorbia Dentata*	0.936	0.134	*Passiflora Foetida*	0.99	0.007
Aeschynomene Americana	0.997	0.004	*Euphorbia Heterophylla*	0.985	0.009	*Passiflora Suberosa*	0.988	0.01
Agave Americana	0.921	0.068	*Euphorbia Hirta*	0.977	0.017	*Pennisetum Polystachion*	0.997	0.003
Ageratina Adenophora	0.98	0.011	*Euphorbia Hypericifolia*	0.988	0.005	*Pennisetum Purpureum*	0.99	0.005
Ageratina Riparia	0.999	0.001	*Euphorbia Maculata*	0.974	0.022	*Peperomia Pellucida*	0.995	0.005
Ageratum Conyzoides	0.971	0.025	*Euphorbia Marginata*	0.991	0.031	*Phyllanthus Tenellus*	0.99	0.01
Ageratum Houstonianum	0.981	0.009	*Euphorbia Nutans*	0.884	0.075	*Physalis Angulata*	0.974	0.015
Agrostemma Githago	0.945	0.081	*Euphorbia Peplus*	0.968	0.022	*Physalis Peruviana*	0.969	0.006
Albizia Lebbeck	0.989	0.004	*Euphorbia Prostrata*	0.987	0.009	*Physalis Pubescens*	0.956	0.008
Alternanthera Paronychioides	0.951	0.002	*Euphorbia Pulcherrima*	0.993	0.002	*Phytolacca Americana*	0.959	0.028
Alternanthera Philoxeroides	0.983	0.011	*Euphorbia Serpens*	0.995	0.003	*Pilea Microphylla*	0.981	0.015
Alternanthera Pungens	0.981	0.042	*Euphorbia Tirucalli*	0.96	0.017	*Pistia Stratiotes*	0.987	0.007
Amaranthus Albus	0.982	0.03	*Evolvulus Nummularius*	0.998	0.002	*Plantago Aristata*	0.986	0.018
Amaranthus Blitoides	0.973	0.062	*Gaillardia Pulchella*	0.967	0.01	*Plantago Virginica*	0.996	0.006
Amaranthus Blitum	0.973	0.007	*Galinsoga Parviflora*	0.951	0.023	*Pluchea Carolinensis*	0.995	0.005
Amaranthus Dubius	0.996	0.002	*Galinsoga Quadriradiata*	0.977	0.012	*Pluchea Sagittalis*	0.994	0.005
Amaranthus Hybridus	0.984	0.005	*Gamochaeta Pensylvanica*	0.989	0.006	*Polygala Paniculata*	0.997	0.003
Amaranthus Palmeri	0.999	0.012	*Gamochaeta Purpurea*	0.997	0.003	*Portulaca Pilosa*	0.993	0.005
Amaranthus Retroflexus	0.951	0.034	*Gaura Parviflora*	0.982	0.037	*Praxelis Clematidea*	0.985	0.012

Amaranthus Spinosus	0.967	0.024	*Geranium Carolinianum*	0.983	0.015	*Pseudelephantopus Spicatus*	0.999	0.002
Amaranthus Viridis	0.978	0.011	*Gomphrena Celosioides*	0.996	0.004	*Ranunculus Muricatus*	0.997	0.004
Ambrosia Artemisiifolia	0.942	0.018	*Gymnocoronis Spilanthoides*	0.997	0.002	*Rhus Typhina*	0.972	0.016
Ambrosia Trifida	0.996	0.014	*Helianthus Tuberosus*	0.949	0.029	*Richardia Brasiliensis*	0.998	0.002
Ammannia Coccinea	0.966	0.006	*Herissantia Crispa*	0.999	0.001	*Richardia Scabra*	0.996	0.004
Anredera Cordifolia	0.984	0.012	*Hibiscus Trionum*	0.939	0.034	*Ricinus Communis*	0.962	0.021
Antigonon Leptopus	0.996	0.004	*Hippobroma Longiflora*	0.983	0.003	*Rivina Humilis*	0.993	0.007
Argemone Mexicana	0.983	0.006	*Hordeum Jubatum*	0.987	0.089	*Robinia Pseudoacacia*	0.946	0.032
Asclepias Curassavica	0.977	0.009	*Hydrocotyle Verticillata*	0.992	0.008	*Salvia Coccinea*	0.987	0.008
Austroeupatorium Inulifolium	0.999	0.002	*Hypochaeris Albiflora*	1	0.001	*Salvinia Molesta*	0.998	0.002
Avena Fatua	0.914	0.037	*Hypochaeris Radicata*	0.999	0.001	*Senecio Vulgaris*	0.941	0.022
Axonopus Compressus	0.993	0.005	*Hyptis Brevipes*	0.996	0.002	*Senna Alata*	0.994	0.006
Azolla Filiculoides	0.979	0.008	*Hyptis Rhomboidea*	0.997	0.004	*Senna Bicapsularis*	0.978	0.014
Begonia Cucullata	0.972	0.004	*Hyptis Suaveolens*	0.995	0.003	*Senna Hirsuta*	0.925	0.006
Bidens Alba	0.975	0.014	*Indigofera Suffruticosa*	0.987	0.01	*Senna Occidentalis*	0.981	0.011
Bidens Bipinnata	0.971	0.027	*Ipomoea Alba*	0.988	0.004	*Senna Sophera*	0.953	0.038
Bidens Frondosa	0.973	0.025	*Ipomoea Cairica*	0.985	0.013	*Sesbania Bispinosa*	0.841	0.026
Bidens Pilosa	0.968	0.014	*Ipomoea Coccinea*	0.861	0.057	*Sesbania Cannabina*	0.99	0.01
Brachiaria Mutica	0.997	0.003	*Ipomoea Hederacea*	0.995	0.004	*Sida Acuta*	0.977	0.005
Bromus Catharticus	0.987	0.005	*Ipomoea Indica*	0.991	0.006	*Silphium Perfoliatum*	0.871	0.082
Bryophyllum Pinnatum	0.981	0.028	*Ipomoea Lacunosa*	0.956	0.049	*Silybum Marianum*	0.856	0.091
Cabomba Caroliniana	0.927	0.071	*Ipomoea Mauritiana*	0.968	0.044	*Solanum Americanum*	0.978	0.015
Cajanus Cajan	0.978	0.011	*Ipomoea Nil*	0.974	0.012	*Solanum Capsicoides*	0.983	0.009
Callisia Repens	0.997	0.003	*Ipomoea Purpurea*	0.96	0.036	*Solanum Diphyllum*	0.991	0.006
Calopogonium Mucunoides	0.993	0.002	*Ipomoea Quamoclit*	0.973	0.019	*Solanum Erianthum*	0.984	0.01
Calyptocarpus Vialis	0.995	0.005	*Ipomoea Triloba*	0.986	0.007	*Solanum Mauritianum*	0.998	0.003
Cannabis Sativa	0.91	0.029	*Iris Pseudacorus*	0.991	0.01	*Solanum Pseudocapsicum*	0.973	0.017
Catharanthus Roseus	0.986	0.01	*Jacquemontia Tamnifolia*	0.999	0.002	*Solanum Scabrum*	0.941	0.015
Cenchrus Echinatus	0.993	0.006	*Kyllinga Polyphylla*	0.996	0.002	*Solanum Sisymbriifolium*	0.96	0.003
Centaurea Cyanus	0.926	0.032	*Lactuca Serriola*	0.894	0.038	*Solanum Torvum*	0.981	0.016
Centrosema Pubescens	0.994	0.005	*Lantana Camara*	0.984	0.015	*Solanum Viarum*	0.951	0.034
Cerastium Glomeratum	0.96	0.019	*Lantana Montevidensis*	0.995	0.005	*Solidago Canadensis*	0.938	0.029
Chamaecrista Mimosoides	0.993	0.006	*Lepidium Densiflorum*	0.967	0.047	*Soliva Anthemifolia*	0.992	0.004
Chenopodium Hybridum	0.905	0.032	*Lepidium Virginicum*	0.982	0.01	*Soliva Sessilis*	0.997	0.003
Chromolaena Odorata	0.988	0.01	*Leucaena Leucocephala*	0.985	0.011	*Sonchus Asper*	0.969	0.009
Cichorium Intybus	0.873	0.139	*Leucanthemum Vulgare*	0.981	0.009	*Sonneratia Apetala*	0.944	0.003
Cleome Rutidosperma	0.989	0.01	*Limnocharis Flava*	0.999	0.002	*Sorghum Halepense*	0.995	0.003
Clitoria Ternatea	0.993	0.006	*Lindernia Rotundifolia*	0.997	0.003	*Spermacoce Remota*	0.994	0.006
Corchorus Olitorius	0.984	0.005	*Lolium Multiflorum*	0.976	0.011	*Sphagneticola Trilobata*	0.988	0.01
Coreopsis Lanceolata	0.942	0.047	*Lolium Perenne*	0.917	0.02	*Stachys Arvensis*	0.992	0.005

Coreopsis Tinctoria	0.887	0.11	*Lolium Temulentum*	0.961	0.102	*Stachytarpheta Cayennensis*	0.997	0.002
Coronilla Varia	0.966	0.096	*Ludwigia Decurrens*	0.987	0.002	*Stachytarpheta Jamaicensis*	0.997	0.004
Coronopus Didymus	0.946	0.015	*Macroptilium Atropurpureum*	0.992	0.008	*Stemodia Verticillata*	0.99	0.005
Cosmos Bipinnatus	0.948	0.032	*Macroptilium Lathyroides*	0.996	0.003	*Stylosanthes Guianensis*	0.952	0.009
Cosmos Sulphureus	0.971	0.016	*Malvastrum Coromandelianum*	0.988	0.009	*Symphyotrichum Subulatum*	0.987	0.01
Crassocephalum Crepidioides	0.966	0.026	*Mecardonia Procumbens*	0.994	0.006	*Symphytum Officinale*	0.908	0.035
Crassocephalum Rubens	0.98	0.022	*Medicago Polymorpha*	0.967	0.015	*Synedrella Nodiflora*	0.992	0.006
Crepis Tectorum	0.922	0.108	*Medicago Sativa*	0.915	0.041	*Tagetes Erecta*	0.924	0.035
Crocosmia	0.979	0.005	*Melilotus Albus*	0.895	0.043	*Talinum Fruticosum*	0.987	0.004
Crotalaria Micans	0.997	0.003	*Melilotus Indicus*	0.972	0.015	*Talinum Paniculatum*	0.98	0.018
Crotalaria Pallida	0.99	0.009	*Melilotus Officinalis*	0.902	0.057	*Taraxacum Officinale*	0.987	0.01
Crotalaria Trichotoma	0.993	0.006	*Melinis Repens*	0.991	0.009	*Tarlmounia Elliptica*	0.998	0.002
Croton Bonplandianus	1	0	*Melothria Pendula*	0.992	0.005	*Tephrosia Candida*	0.993	0.008
Cuphea Carthagenensis	0.992	0.008	*Mikania Micrantha*	0.985	0.012	*Thalia Dealbata*	0.992	0.005
Cuscuta Campestris	0.99	0.005	*Mimosa Bimucronata*	0.981	0.02	*Tithonia Diversifolia*	0.991	0.006
Cyclospermum Leptophyllum	0.99	0.011	*Mimosa Diplotricha*	0.994	0.005	*Tradescantia Fluminensis*	0.993	0.005
Cyperus Esculentus	0.984	0.009	*Mimosa Pigra*	0.999	0.002	*Tradescantia Zebrina*	0.993	0.007
Cyperus Surinamensis	0.971	0.002	*Mimosa Pudica*	0.987	0.012	*Tragopogon Dubius*	0.836	0.111
Datura Innoxia	0.933	0.058	*Mirabilis Jalapa*	0.971	0.019	*Tridax Procumbens*	0.988	0.008
Datura Metel	0.981	0.015	*Mitracarpus Hirtus*	0.992	0.011	*Trifolium Hybridum*	0.972	0.093
Datura Stramonium	0.9	0.044	*Myriophyllum Aquaticum*	0.985	0.01	*Trifolium Pratense*	0.934	0.04
Daucus Carota	0.958	0.027	*Nasturtium Officinale*	0.935	0.029	*Trifolium Repens*	0.948	0.035
Desmodium Tortuosum	0.997	0.003	*Nicandra Physalodes*	0.965	0.02	*Verbena Bonariensis*	0.986	0.008
Dysphania Ambrosioides	0.986	0.004	*Oenothera Biennis*	0.946	0.022	*Verbena Brasiliensis*	0.998	0.002
Egeria Densa	0.993	0.004	*Oenothera Drummondii*	0.998	0.007	*Veronica Arvensis*	0.983	0.011
Eichhornia Crassipes	0.973	0.015	*Oenothera Glazioviana*	0.901	0.021	*Veronica Hederifolia*	0.911	0.045
Elephantopus Tomentosus	0.973	0.028	*Oenothera Laciniata*	0.994	0.003	*Veronica Persica*	0.97	0.025
Emilia Fosbergii	0.998	0.002	*Oenothera Rosea*	0.963	0.019	*Veronica Polita*	0.964	0.015
Emilia Praetermissa	0.995	0.005	*Oenothera Speciosa*	0.995	0.006	*Vicia Villosa*	0.804	0.047
Erechtites Hieraciifolius	0.97	0.009	*Oenothera Tetraptera*	0.972	0.003	*Waltheria Indica*	0.988	0.004
Erechtites Valerianifolius	0.993	0.005	*Oxalis Articulata*	0.989	0.006	*Zephyranthes Carinata*	0.971	0.017
Erigeron Annuus	0.965	0.022	*Oxalis Triangularis*	0.993	0.006	*Zinnia Peruviana*	0.912	0.139
Erigeron Bellioides	0.998	0.002	*Panicum Repens*	0.993	0.006			

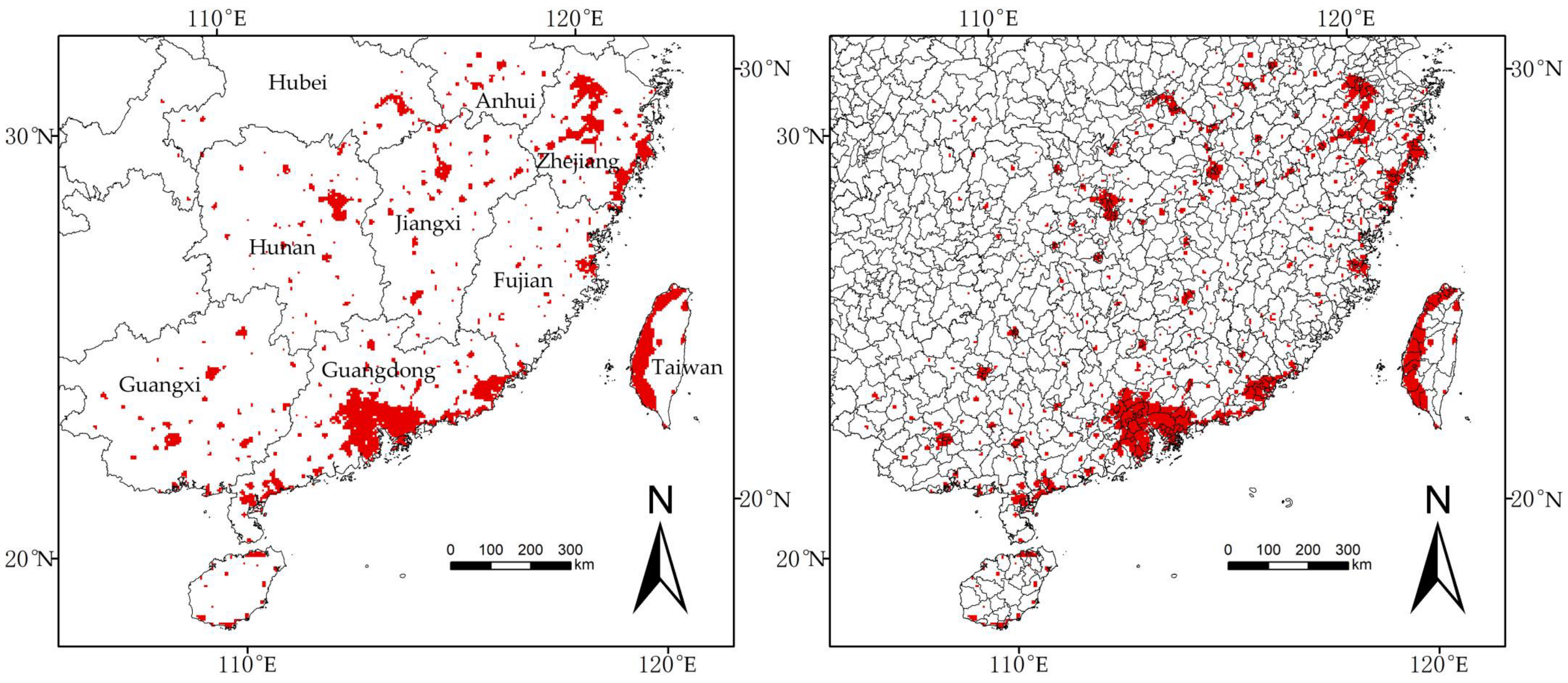

Figure S1 Provincial and county-level distribution of high-risk invasion zones in China.

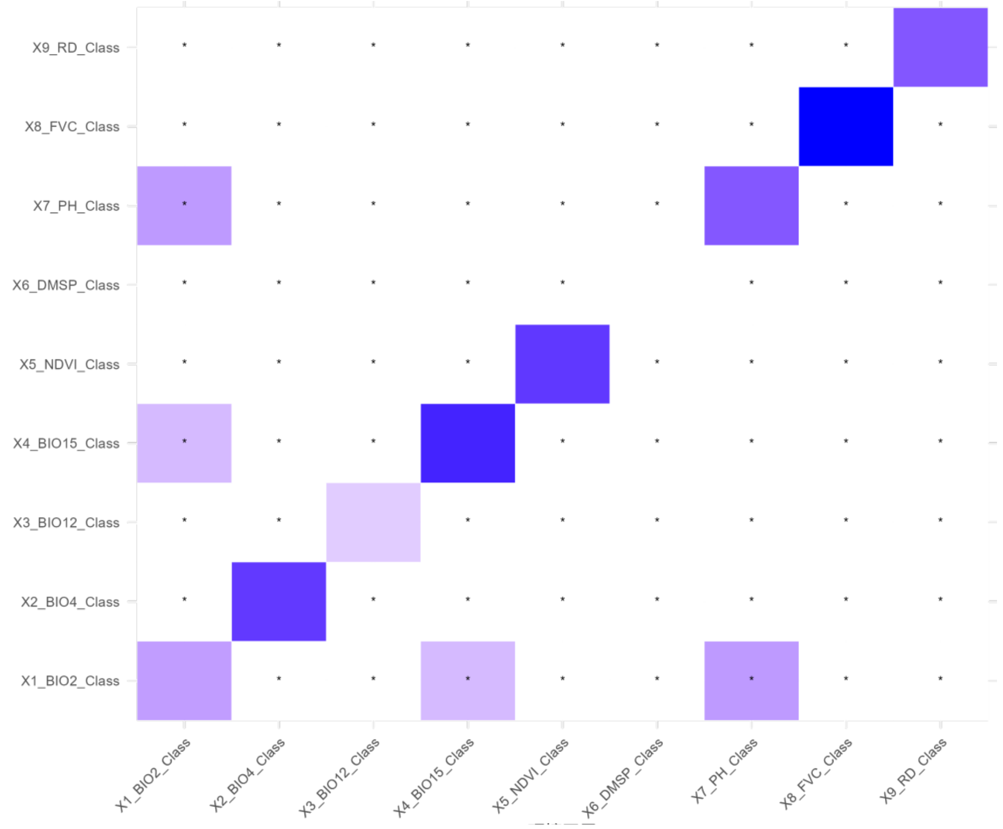

Figure S2 Interaction heatmap of environmental factors.
